# Myocardial scar surface area identified by LGE MRI is an independent predictor of mortality in post-infarction patients

**DOI:** 10.1186/1532-429X-17-S1-P46

**Published:** 2015-02-03

**Authors:** Qian Tao, Sebastiaan R Piers, Hildo J Lamb, Katja Zeppenfeld, Rob J van der Geest

**Affiliations:** 1 Department of Radiology, Leiden University Medical Center, Leiden, Netherlands; 2 Department of Cardiology, Leiden University Medical Center, Leiden, Netherlands

## Background

Recent research has shown that the size of myocardial gray zone (a mixture of normal and infarcted tissue) identified from late gadolinium enhanced (LGE) MRI is an independent predictor of adverse cardiac events in post-infarction patients. However, definition of myocardial gray zone in LGE images (e.g. using the N-STD method) remains difficult for varying MR protocols and qualities, lacks histological validation, and may lead to conflicting conclusions. We sought to establish a novel predictive scar parameter independent of gray zone definition.

## Methods

Eighty-six patients (age 64±10) with prior myocardial infarction underwent a cardiac MRI study prior to ICD implantation. The MRI study included cine-MRI for evaluation of cardiac function e.g. left ventricular ejection fraction (LVEF), and LGE-MRI for assessment of myocardial infarction size and morphology. The total myocardial scar and gray zone sizes were estimated from scar regions identified by the full-width-half-maxima (FWHM) method with 35% and 50% threshold, respectively. The total myocardial scar surface areas were computed based on 3D reconstruction of the identified myocardial scar. Patients were followed at our institute.

## Results

**Figure 1:**
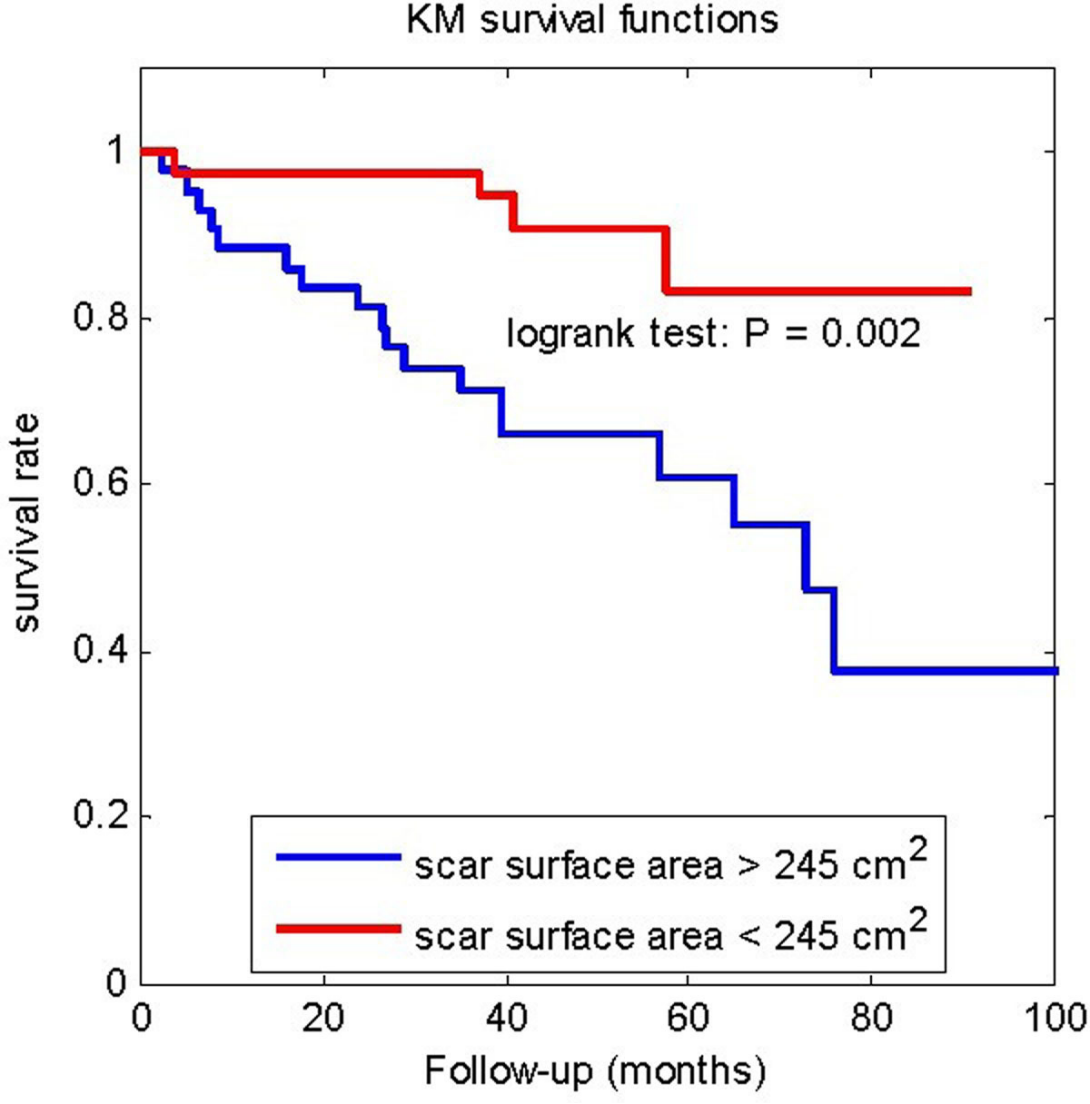
The comparison of estimated Kaplan-Meier survival functions with different scar surface areas. The threshold 245 cm^2^ is the median value of scar surface areas. The P values of logrank test is also reported.

During a median follow-up of 45 months, interquartile 34-58 months, 22 (26%) patients died. Univariate Cox proportional hazard analysis showed that the total scar size, gray zone size, and total scar surface area were all predictors of patient mortality, with a Cox hazard ratio HR=1.16/10g (p=0.01) for total scar size, HR=1.55/10g (p=0.02) for gray zone size, and HR=1.59/100cm^2^ (p=0.003) for total scar surface area. However, after correction for LVEF, multivariate Cox proportional hazard analysis showed that only the total scar surface area remained predictive of all-cause mortality, with HR=1.75/100cm^2^, p=0.01. Table 1 reports the scar sizes, surface areas, HR, and p values. Kaplan-Meier plot of survival functions differentiated by the proposed total scar surface area parameter was presented in Figure 1.

**Table 1:** The results of univariate and multivariate Cox proportional hazard analysis for scar features identified from LGE-MRI.

	Median (IQR)	Univariate Cox hazard analysis			Multivariate Cox hazard analysis (corrected for LVEF)		
		
		HR	95% CI	P value	HR	95% CI	P value
Total scar size	56 g (38-77 g)	1.16/10 g	1.03-1.31	0.01*	1.18/10 g	0.99-1.41	0.06

Gray zone size	14 g (9-23 g)	1.55/10 g	1.06-2.27	0.02*	1.51/10 g	0.94-2.44	0.08

Total scar surface area	245 cm2 (178-334 cm2)	1.59/100 cm2	1.17-2.15	0.003*	1.75/100 cm2	1.13-2.71	0.01*

## Conclusions

Total myocardial scar surface area identified from LGE MRI is a predictor of all-course mortality in post-infarction patients, independent of LVEF. The method used the histologically validated FWHM method to quantify the scar, while avoiding gray zone definition. The study highlights the potential of scar morphology analysis from LGE-MRI, beyond conventional scar size parameters.

## Funding

Dutch Technology Foundation (STW project no.12899).

